# Bio-fertilizer and rotten straw amendments alter the rhizosphere bacterial community and increase oat productivity in a saline–alkaline environment

**DOI:** 10.1038/s41598-020-76978-3

**Published:** 2020-11-16

**Authors:** Peina Lu, Luke D. Bainard, Bin Ma, Jinghui Liu

**Affiliations:** 1 College of Agronomy, Inner Mongolia Agricultural University, Hohhot, 010019 Inner Mongolia China; 2 Swift Current Research and Development Centre, Agriculture and Agri-Food Canada, Swift Current, SK S9H 3X2 Canada; 3 Institute of Desertification Control, Ningxia Academy of Agriculture and Forestry Sciences, Yinchuan, 750002 Ningxia China

**Keywords:** Agroecology, Plant sciences, Bacteria, Microbial ecology

## Abstract

Saline–alkaline conditions can limit crop productivity and the role of soil microbes in nutrient cycling in arid and semi-arid regions throughout the world. A better understanding of how soil amendments and plant varieties affect rhizosphere microbial communities in saline–alkaline environments is important for the development of sustainable and productive agricultural systems under these challenging conditions. The objective of this study was to determine the effect of organic soil amendments on crop yield, soil physicochemical properties and rhizosphere bacterial communities of two oat cultivars in a saline–alkaline soil. The experiment was conducted in a semi-arid region of Northern China and involved growing two oat cultivars with varying levels of saline–alkaline tolerance under four different amendment treatments: (1) control (no amendments), (2) bio-fertilizer, (3) rotten straw, and (4) combination of bio-fertilizer and rotten straw. The combined bio-fertilizer and rotten straw amendment treatment resulted in the highest oat yields, reduced soil pH, and increased soil salt content for both cultivars. Baiyan2 (tolerant cultivar) had a higher bacterial α-diversity, relative abundance of *Proteobacteria* and *Acidobacteria,* and lower relative abundance of *Firmicutes* compared to Caoyou1 (sensitive cultivar). The rotten straw treatment and combined amendment treatment decreased bacterial α-diversity and the abundance of *Proteobacteria*, and increased the abundance of *Firmicutes*, which were positively correlated with soil salt, available nitrogen, phosphorous and potassium for both cultivars. Our study suggested using tolerant oat cultivars with the combined application of rotten straw and bio-fertilizer could be an effective strategy in remediating saline–alkaline soils.

## Introduction

Soil saline–alkalization is a key environmental factor that severely limits the functional roles of soil microbes in arid and semi-arid regions globally^1,2^. It has been suggested that crop management practices such as organic amendments (e.g., bio-fertilizer and rotten straw) are more effective than inorganic amendments (e.g., gypsum^3^) for altering soil nutrient and physiochemical properties, shifting the composition of soil microbial communities and increasing crop yields^4,5^. Bio-fertilizers are natural organic amendments^6–8^, which have been widely used in saline–alkali soils^9^ to improve soil fertility and productivity^10^. Bio-fertilizers refer to the use of inoculants composed of soil microorganisms that promote plant growth by increasing the uptake or availability of primary nutrients to the host plant^11^. Plant straw, a precursor for humus, is a major lignocellulose waste produced in agricultural processes and has been traditionally used for soil amendments in the form of compost. Tan et al.^12^ and Han^13^ found that the decay and nutrient release rate of rotten straw application was faster than direct straw application. In addition, the application of rotten straw has been shown to have a positive effect on soil physicochemical properties (e.g., soil organic carbon and potassium content) and enzyme activity^12,14^. There is increasing evidence that the combined application of bio-fertilizer and rotten straw amendments may be an effective strategy to remediate saline–alkaline soil properties and improve soil productivity in a saline–alkali ecosystem^15,16^.

Applications of bio-fertilizer and rotten straw can potentially alter the bulk and rhizosphere soil environment and microbial community under saline–alkaline conditions. Previous studies have observed that bio-fertilizer application increased the abundance of disease suppressive microorganisms, such as *Acidobacteria* and *Firmicutes*^17,18^, and increase soil bacterial diversity^19–22^. In a long-term experiment, Zhao et al.^23^ found that high rates of maize straw addition altered the structure of soil microbial communities and increased enzyme activities. However, straw additions under controlled conditions were linked to decreased microbial diversity and altered community structures^24^. This shift in the microbial community appears to be linked to the improvement of environmental factors such as soil pH and soil nutrient status, which have been identified as important drivers of bacterial community assembly and diversity in both acid and alkaline conditions^25–29^. Soil enzymes (e.g., catalase, urease, alkaline phosphatase and sucrase) are sensitive to changes in the soil environment and play crucial roles in nutrient cycling and are tightly linked to the bacterial community^27^. Soil microorganisms are essential to the soil environment and play vital roles in soil biogeochemical cycling in agricultural systems^30,31^, and are particularly important in the rhizosphere^32^. As a result, soil pH, salinity and enzyme activities are relevant soil indicators to evaluate the impact that soil amendments have on the soil bacterial community and general soil health in saline–alkaline ecosystems.

Bio-fertilizers and rotten straw amendments have also been shown to benefit plant establishment^32^. However, plant species and genotypes exhibit different responses to soil amendments via enhanced rhizosphere microbial communities^33–38^. For example, alfalfa rhizosphere microbial communities have been shown to differ between genotypes^33,34^, while no differences have been observed in different cultivars of soybean, canola, wheat, rice or maize^35–38^. To this date, there has been no research focused on how soil organic amendments affect the rhizosphere bacterial community structure of plant cultivars with a range of saline–alkaline-tolerance. Oat (*Avena nuda* L.) is considered a pioneer crop for improving saline soil in arid and semi-arid areas because of its saline–alkaline tolerance^39,40^. Different oat genotypes exhibit different saline–alkaline tolerance and productivity under these conditions^41–43^ and it is important to understand how oat varieties with differing saline–alkaline tolerance respond to soil amendments.

We hypothesized that the addition of bio-fertilizer and rotten straw amendments to saline–alkali soil will alter rhizosphere soil physicochemical properties, microbial communities and improve the productivity of oats with different saline–alkaline tolerance. To test this hypothesis, a field experiment was conducted in a typical saline–alkaline region and involved growing two oat cultivars with the following treatments: control (no amendments), bio-fertilizer, rotten straw, and combined bio-fertilizer and rotten straw. The objectives of this study were to analyze the effects of rotten straw and bio-fertilizer on: (1) soil physicochemical properties, (2) enzyme activities, (3) yield of two oat cultivars with different saline–alkali tolerance, and (4) diversity and composition of rhizosphere bacterial communities (Table 1).

**Table 1:** The physicochemical characteristics of the field soil and organic amendments materials.

Parameters	pH	EC	Available N	Available P	Available K	Total N	Total P	Total K	Organic matter
		uS cm^−1^	mg kg^−1^			g kg^−1^			
Experimental field	9.14	1553.83	63.11	15.71	171.33	0.53	1.66	0.53	13.32
Bio-fertilizer	6.85	125.93	532.42	156.52	222.79	84.67	0.16	9.33	372.60
Rotten straw	6.58	2.95	362.06	169.70	345.39	14.06	0.18	7.99	552.48

## Results

### Oat productivity

**Figure 1:**
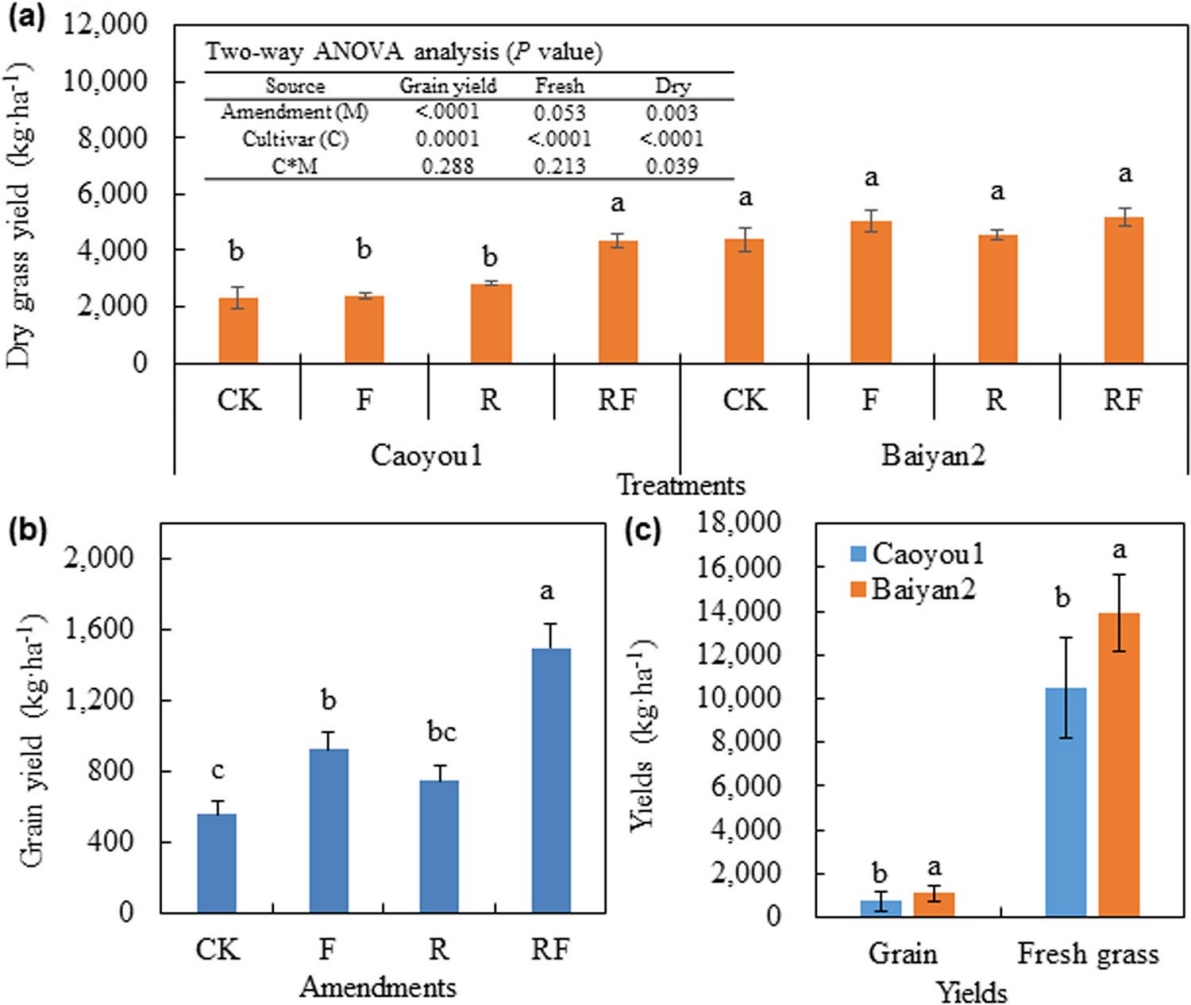
Effect of soil amendments on grain yield and grass biomass production of two oat cultivars. CK was negative control; F was application of bio-fertilizer; R was application of rotten straw; RF was co-application of bio-fertilizer and rotten straw.

The grain, dry grass and fresh grass yields of Baiyan2 was significantly higher than Caoyou1 (Fig. 1). The bio-fertilizer amendment significantly increased the grain yield of both cultivars, but maximum yields were observed with the combined application of bio-fertilizer and rotten straw. There was no effect of the amendments on the fresh and dry grass yields of the Baiyan2 cultivar. However, the combined amendment treatment significantly increased the grass yield for Caoyou1, which produced similar yields as the Baiyan2 cultivar.

### Rhizosphere soil properties

Compared to the control, the combined amendment treatment significantly reduced soil pH, increased soil water content, but also led to higher salt content (Table 2). Rotten straw significantly increased rhizosphere soil available potassium (AK), available phosphorus (AP) and available nitrogen (AN) for both cultivars, whereas the bio-fertilizer had no significant effect on these soil nutrients compared to the control. Cultivar had significant effect on AP content as Baiyan2 had a significantly higher AP content compared to Caoyou1.

**Table 2:** Effect of amendments on rhizosphere soil properties of Caoyou1 (A) and Baiyan2 (B) oat cultivars.

Treatments	SW (%)	pH	Salt (%)	AK (mg kg^−1^)	AP (mg kg^−1^)	AN (mg kg^−1^)	Catalase (mL of 0.1 mol L^−1^ KMnO_4_ g^−1^ soil 30 min^−1^)	Alkaline phosphate (mg phenol g^−1^ soil 24 h^−1^)	Urease (mg NH_4_-N g^−1^ soil 24 h^−1^)	Sucrase (mg glucose g^−1^ soil 24 h^−1^)
**Cultivar (C)**										
Caoyou1(A)	5.95 ± 0.17b	8.02 ± 0.02	0.56 ± 0.02	135.17 ± 13.75	38.23 ± 4.7b	27.64 ± 2.83	8.74 ± 0.54	25.33 ± 1.27a	1.18 ± 0.05	50.45 ± 2.14b
Baiyan2(B)	7.69 ± 0.93a	8.02 ± 0.03	0.59 ± 0.04	142.50 ± 22.40	47.32 ± 4.44a	26.00 ± 3.11	8.97 ± 0.56	22.49 ± 0.83b	1.18 ± 0.05	65.26 ± 4.78a
**Amendment (M)**										
CK	5.68 ± 0.10b	8.11 ± 0.01a	0.47 ± 0c	71.67 ± 10.06b	27.39 ± 01c	17.02 ± 1.59c	7.08 ± 0.48b	21.69 ± 1.13b	1.26 ± 0.05a	49.38 ± 3.68b
F	5.60 ± 0.29b	7.96 ± 0.04b	0.53 ± 0.01bc	92.00 ± 4.47b	29.32 ± 04c	21.93 ± 2.53bc	9.21 ± 0.44a	25.49 ± 2.94a	1.23 ± 0.07a	53.25 ± 3.54b
R	6.73 ± 0.55b	8.06 ± 0.02a	0.56 ± 0.01b	192.50 ± 10.39a	52.95 ± 02b	27.55 ± 1.35b	10.4 ± 0.17a	24.42 ± 0.26a	1.22 ± 0.06a	72.26 ± 8.19a
RF	9.26 ± 1.55a	7.94 ± 0.01b	0.75 ± 0.04a	199.17 ± 13.38a	61.44 ± 01a	40.78 ± 2.15a	8.76 ± 1.05a	24.03 ± 0.37a	1.01 ± 0.04b	56.51 ± 3.32b
C*M										
A1	5.71 ± 0.19bc	8.08 ± 0.01bc	0.48 ± 0de	90 ± 12.58c	23.14 ± 0.61d	15.45 ± 2.75	7.73 ± 0.27c	22.24 ± 1.72 cd	1.21 ± 0.09	41.92 ± 0.10
A2	6.01 ± 0.02bc	8.05 ± 0c	0.55 ± 0 cd	95.67 ± 0.67c	24.98 ± 1.67d	24.75 ± 2.51	10.19 ± 0.06a	31.77 ± 1.94a	1.17 ± 0.14	52.11 ± 3.94
A3	6.05 ± 0.74bc	8.01 ± 0d	0.53 ± 0cde	183.33 ± 18.33b	43.44 ± 0.78b	30.15 ± 1.30	10.04 ± 0.09ab	23.95 ± 0.35bc	1.29 ± 0.10	56.84 ± 1.84
A4	6.03 ± 0.01bc	7.93 ± 0.03e	0.69 ± 0b	171.67 ± 6.01b	61.35 ± 0.42a	40.20 ± 0.75	7.01 ± 1.57c	23.34 ± 0.19bc	1.03 ± 0.05	50.91 ± 4.86
B1	5.65 ± 0.12bc	8.14 ± 0a	0.46 ± 0e	53.33 ± 3.33d	31.64 ± 0.26c	18.60 ± 1.62	6.42 ± 0.81c	21.14 ± 1.78 cd	1.31 ± 0.02	56.84 ± 3.48
B2	5.19 ± 0.51c	7.88 ± 0.01f.	0.51 ± 0de	88.33 ± 9.28c	33.66 ± 0.91c	19.10 ± 4.21	8.22 ± 0.07bc	19.22 ± 0.10d	1.28 ± 0.06	54.39 ± 6.76
B3	7.42 ± 0.71b	8.12 ± 0ab	0.59 ± 0c	201.67 ± 10.93ab	62.45 ± 0.21a	24.95 ± 0.78	10.76 ± 0.12a	24.89 ± 0.06b	1.16 ± 0.05	87.69 ± 9.71
B4	12.50 ± 1.21a	7.95 ± 0e	0.81 ± 0.07a	226.67 ± 10.14a	61.53 ± 0.32a	41.35 ± 4.71	10.5 ± 0.12a	24.72 ± 0.41b	0.98 ± 0.08	62.10 ± 0.28
**ANOVA table (LSD protected,** ***P*** **≤ 0.05)**										
Cultivar	< 0.0001	0.607	0.129	0.361	< 0.0001	0.433	0.626	0.001	0.923	0.001
Amendment	< 0.0001	< 0.0001	< 0.0001	< 0.0001	< 0.0001	< 0.0001	0.002	0.014	0.033	0.002
C*M	< 0.0001	< 0.0001	0.025	0.006	< 0.0001	0.344	0.004	< 0.0001	0.426	0.067

CK (A1 and B1) was negative control; F (A2 and B2) was bio-fertilizer treatment; R (A3 and B3) was rotten straw treatment; RF (A4 and B4) was bio-fertilizer + rotten straw treatment. Values were represented as means ± SEs, and the different small letters within each column of cultivar (C), amendment (M) and C*M means significantly differences at 0.05 level based on ANOVA test.

Catalase and alkaline phosphatase activity were significantly increased by the rotten straw amendment for Baiyan2, while significantly increased by the bio-fertilizer amendment for Caoyou1. For both cultivars, the rotten straw amendment significantly increased sucrase activity, and the combined amendment treatment significantly decreased urease activity.

### Rhizosphere bacterial community

**Figure 2:**
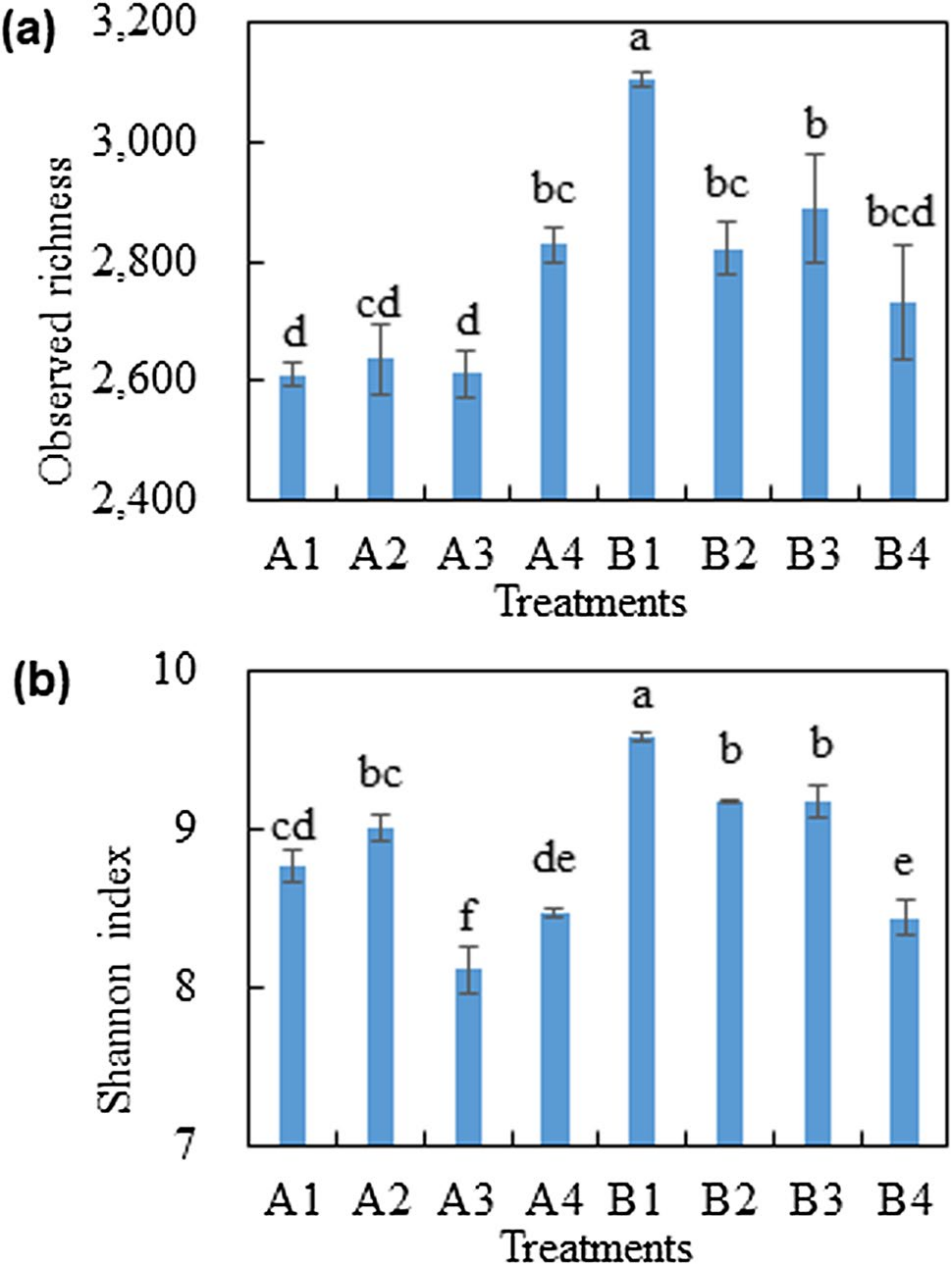
Effect of soil amendments on soil bacterial α-diversity in the rhizosphere of Caoyou1 (**a**) and Baiyan2 (**b**) oat cultivars. A1 and B1 were negative control; A2 and B2 were application of bio-fertilizer; A3 and B3 were application of rotten straw; A4 and B4 were the combined application of bio-fertilizer and rotten straw. The same as below.

**Figure 3:**
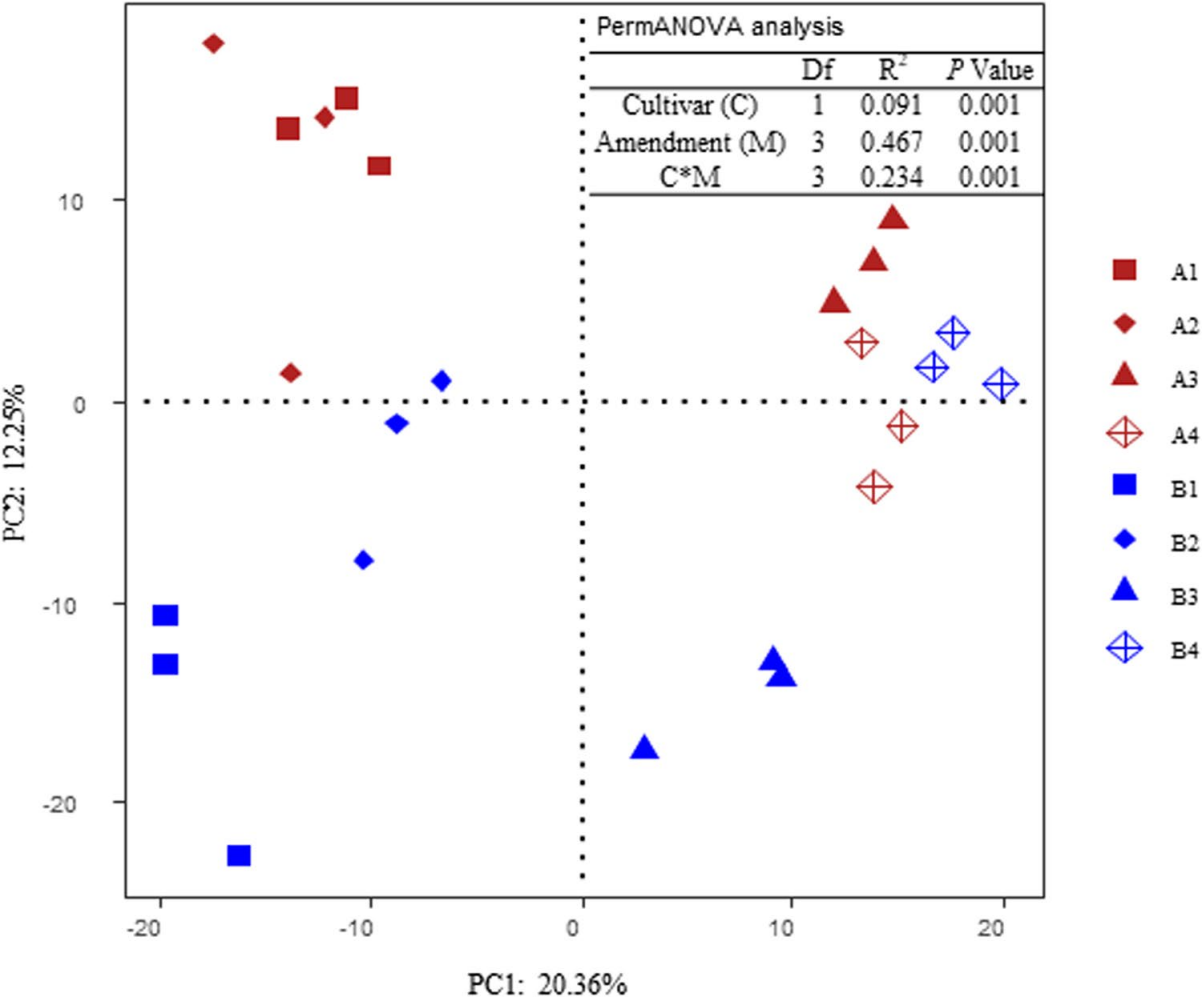
Principal component analysis (PCA) of the rhizosphere soil bacterial community associated with the two oat cultivars and organic soil amendment treatments.

All of the organic amendment treatments significantly reduced the observed richness of the rhizosphere bacterial community associated with Baiyan2, whereas the combined amendment treatment significantly increased the observed richness of the rhizosphere bacterial community associated with Cayou1 (Fig. 2 and Table 3). Furthermore, cultivar significantly affected the estimated richness (Chao1) and diversity (Shannon index) of the rhizosphere bacterial community, with Baiyan2 exhibiting higher levels than Caoyou1 (Table 3). Amendment treatments that included rotten straw significantly decreased the rhizosphere bacterial diversity for Cayou1, and the combined amendment treatment significantly decreased the bacterial diversity for Baiyan2. The permanova and PCA results revealed that cultivar (C), amendment (M) and C × M interactions significantly affected the rhizosphere bacterial community composition (Fig. 3). In particular, the rhizosphere bacterial community associated with amendment treatments that included rotten straw were distinct and separated along the first axis (PC1, Fig. 3) from those associated with treatments that did not include rotten straw (Fig. 3). The rhizosphere bacterial community associated with each cultivar were distinct and separated along the second axis (PC2), with the exception of the combined amendment treatment where both cultivars clustered together.

**Table 3:** Effect of amendments on rhizosphere bacterial α-diversity including community richness and diversity of Caoyou1 and Baiyan2 oat cultivars.

Treatments	Observed richness	Chao1	Shannon
**Cultivar (C)**			
Caoyou1	2,671 ± 34	3,169 ± 70b	8.58 ± 0.11b
Baiyan2	2,887 ± 56	3,413 ± 112a	9.09 ± 0.13a
**Amendment (M)**			
CK	2,858 ± 79a	3,406 ± 138	9.17 ± 0.14a
F	2,729 ± 41b	3,119 ± 43	9.08 ± 0.04ab
R	2,749 ± 58b	3,254 ± 90	8.64 ± 0.18bc
RF	2,780 ± 42b	3,385 ± 99	8.45 ± 0.04c
**ANOVA table (LSD protected,** ***P*** **≤ 0.05)**			
Cultivar (C)	0.322	0.004	< 0.0001
Amendment (M)	0.001	0.061	< 0.0001
C*M	0.008	0.086	< 0.0001

CK was negative control; F was bio-fertilizer treatment; R was rotten straw treatment; RF was bio-fertilizer + rotten straw treatment. Values were represented as means ± SEs, and the different small letters within each column of cultivar (C), amendment (M) and C*M means significantly differences at 0.05 level based on ANOVA test.

**Figure 4:**
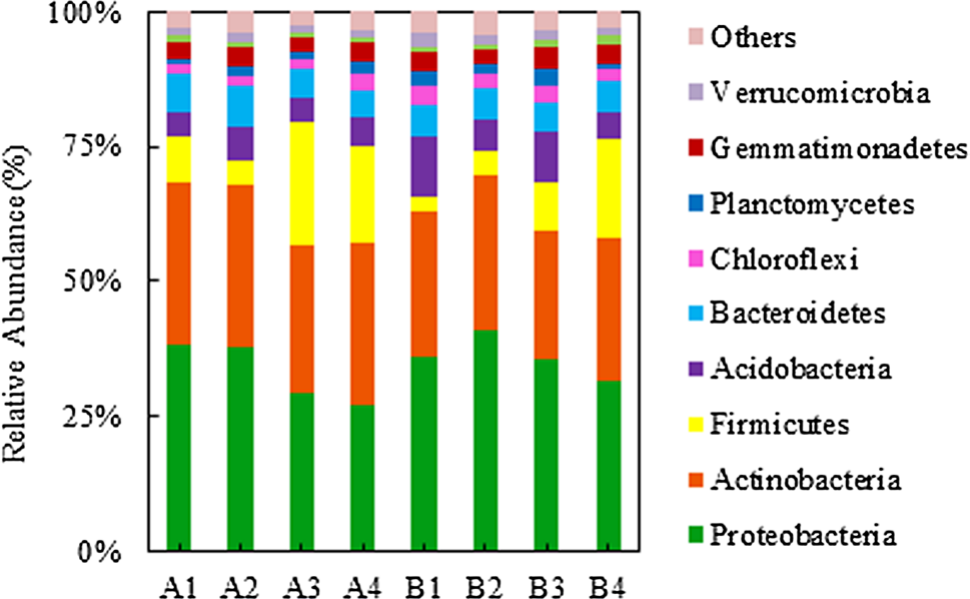
Effect of organic soil amendments on the rhizosphere bacterial community structure at the phylum level.

The dominant rhizosphere bacterial phyla were *Proteobacteria* (27.28–41.09%), *Actinobacteria* (24.17–30.36%) and *Firmicutes* (2.82–22.95%) across all treatments (Fig. 4). Baiyan2 had a higher abundance of *Proteobacteria* (i.e. *Azotobacter*, *Massilia Pseudomonas*) and lower the abundance of *Firmicutes* than Caoyou1 (Table S2). Rotten straw significantly increased the abundance of *Firmicutes*, and decreased the abundance of *Proteobacteria* and genera *Massilia* and *Nocardioides* (Fig. 4, Tables S2 and S3). In contrast, the bio-fertilizer significantly increased *Massilia* and *Nocardioides* for Caoyou1, and significantly increased *Azotobacter* and *Pseudomonas* for Baiyan2 (Table S3).

### Correlation analysis

**Figure 5:**
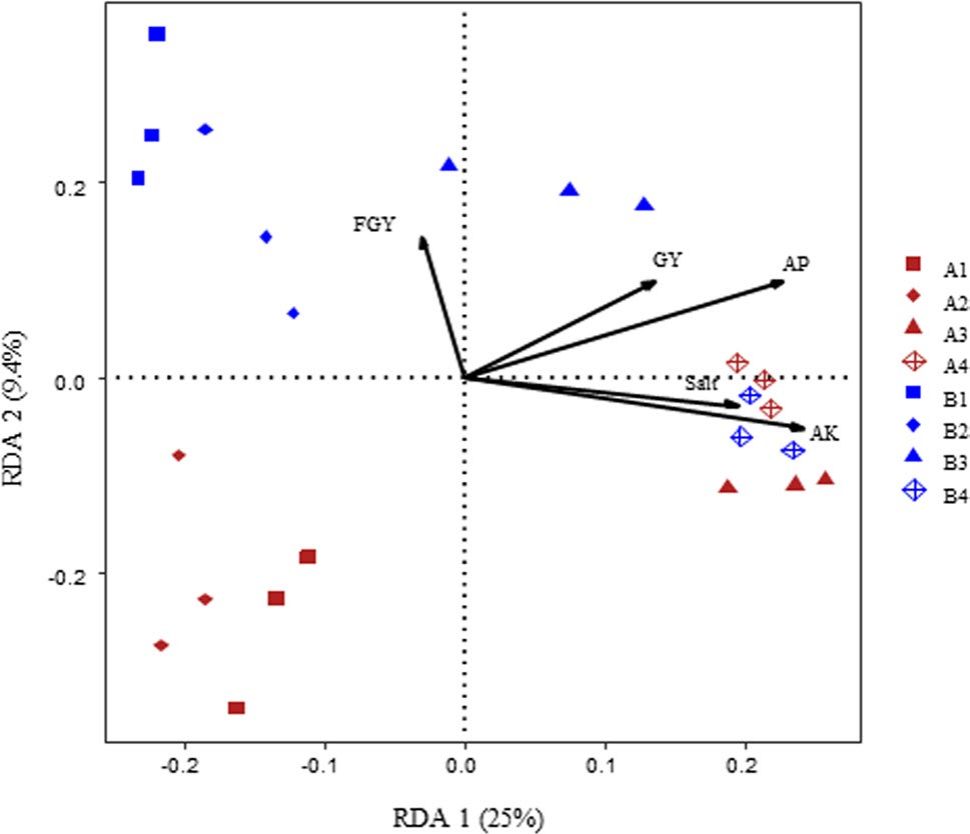
Redundancy analysis (RDA) of the rhizosphere bacterial community, soil properties and oat productivity measurements of both cultivars in different amendment treatments. The parameters were: *GY* grain yield, *FGY* fresh grass yield, *Salt* soil salt content, *AK* soil available potassium, *AP* soil available phosphorus.

The RDA results revealed that the oat rhizosphere bacterial communities associated with treatments that included rotten straw were positively related to soil salt, AK, AP and grass yield for both cultivars (Fig. 5 and Table S4). Spearman correlation analysis showed that soil salt, AK, AP and AN were positively correlated with the relative abundance of *Bacillus, Pseudarthrobacter* and *Planomicrobium*, while negatively correlated with the relative abundance of *Sphingomonas*, *Massilia, Nocardioides* and *Pseudomonas*. Oat yield and ALP were negatively correlated with the relative abundance of *Massilia* (Fig. S1). Grain yield, soil salt, AK, AP, and AN were positively correlated with each other, and negatively correlated with soil pH and urease.

## Discussion

### Change in oat productivity and rhizosphere soil environment

The combined application of bio-fertilizer and rotten straw resulted in the highest oat productivity, particularly in respect to grain yield and dry biomass production. Turmuktini et al.^15^ also reported that the combination of composted straw and biological fertilizer significantly increased rice yield. More specifically, we found that the combined amendment treatment was able to increase grain yield and biomass productivity of the saline–alkaline sensitive oat cultivar to comparable levels of the tolerant cultivar. Reduction of soil pH and promotion of nutrients in the rhizosphere appear to be linked to increased oat productivity. One of the drawbacks of the combined amendment treatment was the higher salt content of the soil. This was possibly due to the combined organic materials containing higher salt content than the bio-fertilizer or rotten straw applications alone^44^. In addition, the soil salt content moved with the moisture and the preservation of soil moisture likely promoted the retention of salt^45^.

In our study, the addition of rotten straw increased the levels of available nutrients (N, P, K) in the rhizosphere soil, which appears to be linked to the added nutrients from these amendment materials. Tan, et al.^12^ found that saline alkaline soil nutrients were directly improved when rotten straw was added due to the quick release of nutrients. The other reason was linked to the greater reduction in soil pH with bio-fertilizer and rotten straw addition, which may increase the availability of N, P and K in solution^46^. Overall, our results indicated that the combined application of rotten straw and bio-fertilizer was an effective management tool for improving soil nutrient availability and productivity of oats in saline–alkaline soils.

### Shift in rhizosphere bacterial community

Our study demonstrated that the soil organic amendments had a contrasting effect on the rhizosphere bacterial community associated with the oat cultivars (i.e., richness decreased in Baiyan2 and increased and Cayou1), with Baiyan2 exhibiting a higher overall richness compared to Caoyou1 under saline–alkaline conditions. Our observations showed that the addition of bio-fertilizer alone significantly increased *Proteobacteria Massilia* for Caoyou1 and increased *Proteobacteria* (i.e. *Azotobacter* and *Pseudomonas*) for Baiyan2, which may be caused by lower soil salt content and available nutrients in this study. Previous studies observed that the application of bio-fertilizer increased the abundance of beneficial microorganisms such as *Azotobacter* that can increase crop yields^49,50^.

The application of rotten straw induced a strong shift in the composition of the rhizosphere bacterial community associated with both oat cultivars. More specifically, rotten straw decreased the abundance of *Proteobacteria* (i.e. *Massilia*) and *Actinobacteria* (i.e.,* Nocardioides*), which were negatively correlated with soil salt, and increased the relative abundance of *Firmicutes* (i.e. *Bacillus* and *Planomicrobium*), which was positively correlated with AK, AP and AN in this study. Other studies have also shown that rotten straw can cause higher N availability and/or lower C/N litter input^51^ and higher salinity in the soil that can lead to lower abundance of *Proteobacteria* and *Actinobacteria*^52^. *Massilia* and *Nocardioides* were widely reduced in the process of plant litter decomposition and may be driven by shifts in nutrient availability or pH conditions^53,54^. Similar to Zhao et al.^23^ and Sun et al.^55^, the relative abundance of *Firmicutes* was increased and *Acidobacteria* was decreased by straw addition. Zhao et al.^29^ reported that K^+^ ions play a critical role in promoting bacterial growth (e.g. *Firmicutes*) as the protons are replaced to cope with high external Na^+^ stress. Thus, the application of rotten straw promoted beneficial microbial taxa such as *Firmicutes* and enhanced soil enzyme activities main caused by shifted soil nutrients, which are good for soil health^56^.

Rhizosphere bacteria are essential component of the soil environment and play vital roles in soil–plant systems^30,31^, which are affected by crop cultivar genotypes^33,34^. Our results showed that Baiyan2 (tolerant cultivar) had higher bacterial richness and diversity than Caoyou1 (sensitive cultivar). The rhizosphere bacterial community associated with Baiyan2 was characterized by a higher relative abundance of beneficial *Proteobacteria* genera (i.e. *Azotobacter*, *Massilia* and *Pseudomonas*^49,50^) and lower relative abundance of *Firmicutes* (i.e. *Bacillus*) compared to Caoyou1. This difference may be due to variation of root exudates produced by the two oat cultivars, as root exudate profiles have been shown to vary among varieties and cultivars of the same species^57,58^.

Compared the bacterial communities appeared in soil amendments (bio-fertilizer and rotten straw) with that detected in different treatment soils, our data revealed that soil organic amendments appear to alter the rhizosphere bacterial communities primarily via changes to the soil physicochemical properties, rather than the direct input of exogenous bacterial species from organic materials in the saline–alkali soil. It was reported that most bacteria in the organic materials are adapted to their environments and are likely less competitive than indigenous bacterial species in the soil^47,48^.

### Altered rhizosphere soil enzyme activity

Soil enzymes are involved in the biological cycling of carbon, phosphorus and nitrogen in soil^23,59,60^ and are regarded as potential indicators of soil nutrient cycling^2,61^. The improved enzyme activities in rhizosphere soil from our study could be attributed to the exogenous addition of enzymes from the organic amendments^56^, from the contributions of soil microorganisms^62^, and/or a result of the increased C and N substrate availability^2,4,61,63^. Rotten straw and bio-fertilizer improved soil catalase, alkaline phosphatase and sucrase activities for both cultivars, which all were related to bacterial communities, but had no significant effect on oat yields in this study. Shi et al.^27^ showed that soil enzymes are sensitive to changes in the soil environment and are tightly linked to the bacterial community. However, the combination of bio-fertilizer and rotten straw inhibited urease activity in saline–alkaline soil, which may be due to the higher salt content in rhizosphere soil or addition of N from organic materials^64^. The reduced urease activities in the combined amendment treatment may have been a limiting factor in maximizing the oat productivity.

### Mechanisms of improving oat productivity by bio-fertilizer and rotten straw amendments

In the saline–alkaline environment, the productivity of both oat cultivars were improved through the application of the bio-fertilizer, rotten straw and the combined amendment treatments. This was primarily linked to a reduction in soil pH, increased availability of nutrients, and shift in the rhizosphere bacterial community and enzyme activities. With the process of soil health improvement at this study site, Baiyan2, a tolerant cultivar, has a higher relative abundance of potentially beneficial bacteria (i.e., *Azotobacter* and *Pseudomonas*), which would then foster greater crop yields than Caoyou1^49,50^. Although Caoyou1 is a sensitive cultivar, the oat yields and soil nutrients significantly improved along with a shift in the rhizosphere bacterial community following the application of rotten straw and bio-fertilizer. However, it is important to consider a tolerant cultivar with the highest yields to avoid increasing salinity issues through the application of organic amendment in saline–alkaline land. Our study indicates that tolerant crops with the combination of rotten straw and bio-fertilizer applications should be an effective strategy in remediating saline–alkaline land in semi-arid regions, which could be further investigated in long-term studies.

## Materials and methods

### Study site

The field experiment was conducted from 2016 to 2017 in the Tumote Zuoqi Hailiu village (East 111°22′30″, Latitude 40°41′30″) of Hohhot, Inner Mongolia, China. At the experimental site, the mean annual temperature is 13.2 °C and mean annual rainfall is 410 mm. Soil samples were collected at the beginning of the experiment in April 2016 to determine the baseline soil physicochemical properties (Table 1).

### Characterization of organic soil amendments

Rotten straw was made using corn straw that was cut into pieces of 5 cm in length and fermented without oxygen from Sep. 2015 to Apr. 2016 and from Sep. 2016 to Apr. 2017 for the 2016 and 2017 experiments, respectively. Physicochemical properties of the bio-fertilizer and the rotten straw were determined using the same methods as described for the soil samples below.

### Experimental design and management

The experiment was designed as a 4 × 2 factorial experiment in which the 8 treatments were arranged in a randomized complete block design with three replications for each year (2016 and 2017). Each block had an area of 5 m × 4 m. A (Caoyou1, saline–alkaline sensitive cultivar) and B (Baiyan2, saline–alkaline tolerant cultivar) represented two oat cultivars with different saline–alkaline tolerance^41^. The treatments were as follows: A1 and B1 (no amendments); A2 and B2 (1500 kg ha^−1^ year^−1^ bio-fertilizer); A3 and B3 (12,000 kg ha^−1^ year^−1^ rotten straw); A4 and B4 (combined 1500 kg ha^−1^ year^−1^ bio-fertilizer and 12 000 ha^−1^ year^−1^ rotten straw). Oats were seeded at a rate of 150 kg ha^−1^ year^−1^ and row spacing of 25 cm. Diammonium phosphate was applied at a rate of 150 kg ha^−1^ year^−1^ (DAP: 18-46-0) as the basal fertilizer for each plot. Bio-fertilizer and rotten straw were broadcast uniformly on the soil surface and incorporated into the soil (about 15 cm) by cultivation with a rotary tiller before sowing.

### Field and laboratory measurements

Soil samples were randomly taken from three points within each block at the pre-seeding and heading stages. Rhizosphere soil was collected by excavating oat plants along 50 cm of a row by gently shaking off the loose soil and retaining the soil within 2 mm of the oat root using a bristle brush. Each sample was sieved through a 2 mm mesh screen and divided into three subsamples: fresh soil was stored at 4 °C, air-dried for soil physicochemical and enzyme activity assays, and soil for DNA extraction stored at − 80 °C.

### Determination of the soil physicochemical properties

Soil water content (SW, %) was determined by oven-drying at 105 °C until stable weight and calculated according to the following formula: SW = (soil fresh weight − soil dry weight)/soil dry weight*100. Soil pH was determined by pH meter (STARTER3100) at a ratio of 1:5 for soil to RO water. The soil salt content (Salt) was determine using an electrical conductivity meter (STARTER3100C) at a ratio of 1:2.5 for soil to RO water. Soil available potassium (AK) was determined in 1 M of ammonium acetate extracts by flame photometer (FP6410). Soil available phosphorus (AP) was extracted with 0.5 M NaHCO_3_ and determined using the molybdenum blue method^65^. Soil available nitrogen (AN) was determined by diffusion methods^66^.

### Determination of the soil enzyme activities

Catalase activity was assayed by the potassium permanganate titration method^67–69^. Alkaline phosphatase activity was assayed by the disodium phenyl phosphate method^67,68,70^. Urease activity was assayed by the colorimetric method^67,68,71^. Sucrase activity was assayed with the 3,5-dinitrosalicylic acid method^67,72^.

### High-throughput sequencing

Soil DNA Isolation Kit (MO BIO Laboratories, Inc., Carlsbad, CA, USA) was used to extract DNA from fresh soil samples following the manufacturer's protocol. 1% agarose gel (1% AGE, 100 V/40 min) was used to test the concentrations and purities of DNA and then stored at − 80 °C until analysis. The libraries for the V4 region of bacterial 16S rRNA gene using the primer set of 515F (5′-GTGCCAGCMGCCGCGGTAA-3′) and 806R (5′-GGACTACVSGGGTATCTAAT-3′)^73^ were prepared and sequenced on an Illumina HiSeq2500 platform by Novogene Bioinformatics Technology Co. Ltd (Beijing, China).

Raw sequences were divided into sample libraries using unique barcodes and trimmed to remove the primers and barcodes. Raw sequences were merged using FLASH^74^ and processed using Qiime^75^. Chimeric sequences were removed using UCHIME by comparing with the reference Gold database^76^. OTUs (Operational Taxonomic Units) were clustered at 97% identity and the most abundant sequence in each OTU was selected as a representative sequence using UPARSE software^77^. For each representative sequence, the bacterial 16S database was classified using the SILVA database^78^ and RDP classifier^79^ algorithm to annotate taxonomic information.

### Statistical analysis

Oat productivity (grain yield, dry and fresh biomass production), soil properties and bacterial α-diversity (observed richness, Chao1 richness estimator, Shannon diversity index) were evaluated using the general linear models (GLM) procedure and significant differences among means were separated using Fisher's least significant difference (F-LSD) at 5% level by SAS 9.0^80^. Permutational multivariate analysis of variance based on Bray–Curtis dissimilarity matrices using the “adonis” function in R and principal component analysis (PCA) were used to test and visualize cultivar and amendment treatment effects on the rhizosphere bacterial communities using the R packages “vegan”, “pairwise”, “ade4” and “ggplot2”. Soil properties and oat productivity measurements with significant effects (*P* < 0.05) were kept for redundancy analysis (RDA), excluding collinear variables with a variance inflation factor (VIF > 10) using the “vif.cca” function in R. RDA was used to evaluate the relationships between bacterial communities, oat productivity and soil properties using the “rda” and “ggplot2” function in R^81,82^. In addition, the relationships among bacterial communities, soil properties and oat productivity were examined by performing Spearman’s correlations, and relationships between soil properties and oat productivity were analyzed by performing Pearson correlation analysis.

## Supplementary information

Supplementary Information.

## Data Availability

The datasets generated during and/or analyzed during the current study are available from the corresponding author on reasonable request.
