# Supplementary material for: Bio-fertilizer and rotten straw amendments alter the rhizosphere bacterial community and increase oat productivity in a saline–alkaline environment
Source: Sci Rep. 2020 Nov 16;10:19896. doi: 10.1038/s41598-020-76978-3 (PMC7669890; doi:10.1038/s41598-020-76978-3)
Supplement: Supplementary file 1 — Supplementary Information. [file 41598_2020_76978_MOESM1_ESM.pdf]

Bio-fertilizer and rotten straw amendments alter the rhizosphere bacterial community and increase oat productivity in a saline-alkaline environment

Peina Lu[1,2], Luke D. Bainard[2], Bin Ma[3], Jinghui Liu[1*]

[1]College of Agronomy, Inner Mongolia Agricultural University, Hohhot, Inner Mongolia 010019, China

[2]Swift Current Research and Development Centre, Agriculture and Agri-Food Canada, Swift Current, SK S9H 3X2, Canada

[3] Institute of Desertification Control, Ningxia Academy of Agriculture and Forestry Sciences, Yinchuan, Ningxia 750002, China

Peina Lu: peina-lu@hotmail.com

Luke D. Bainard: luke.bainard@canada.ca

Bin Ma: mbin89@163.com

*** Corresponding author: Jinghui Liu:** cauljh@aliyun.com; Tel: +86-13848150459

Table S1. Bacterial composition of the soil amendments

Phylum	Rotten straw (%)	Bio-fertilizer (%)	Species	Rotten straw (%)	Bio-fertilizer (%)
Proteobacteria	78.61	62.77	Dysgonomonas_mossii	0.01	9.42
Firmicutes	20.95	15.76	Clostridium_sp._KNHs205	2.10	0.02
Bacteroidetes	0.03	18.36	Burkholderia_sp._CCGE1003	0.01	1.55
Actinobacteria	0.02	1.01	Pseudogymnoascus_pannorum	0.77	0.00
Cyanobacteria	0.00	0.76	Lactobacillus_iners	0.00	0.62
Tenericutes	0.39	0.23	Clostridium_sp._KNHs209	0.61	0.17
Acidobacteria	0.00	0.31	Anoxybacillus_toebii	0.00	0.49
Gemmatimonadetes	0.00	0.31	Lysinibacillus_varians	0.14	0.47
Chloroflexi	0.00	0.10	Marinobacter_hydrocarbonoclasticus	0.00	0.42
Nitrospirae	0.00	0.05	[Pseudomonas]_geniculata	0.01	0.37
Others	0.00	0.33	Others	96.35	86.48

Table S2. The related abundance (percentage of reads) and interaction effect analysis of bacteria at the phylum level. A and B were representing the Caoyou1 and Baiyan2 oat cultivars, respectively. CK (A1 and B1) was negative control; F (A2 and B2) was bio-fertilizer treatment; R (A3 and B3) was rotten straw treatment; RF (A4 and B4) was bio-fertilizer + rotten straw treatment. Values were represented as means ± SEs, and the different small letters within each column of cultivar (C), amendment (M) and C*M means significantly differences at 0.05 level based on ANOVA test.

	Proteobacteria	Actinobacteria	Firmicutes	Acidobacteria	Bacteroidetes	Chloroflexi	Planctomycetes	Gemmatimonadetes	Verrucomicrobia
Cultivar (C)									
Caoyou1(A)	33.11b	29.43	13.46a	5.17b	6.10	2.34	1.56b	3.30	1.38b
Baiyan2(B)	36.10a	26.58	8.63b	7.79a	5.59	2.83	2.28a	3.55	1.90a
Amendment (M)									
CK	74.41a	57.03	11.18b	15.80a	12.78	5.28	3.90	6.93	3.66
F	78.78a	59.16	8.91b	12.18ab	12.91	4.62	3.59	6.50	3.40
R	64.57b	51.69	31.82a	13.90a	10.34	5.17	4.43	7.07	3.43
RF	59.07b	56.14	36.45a	9.93b	10.77	5.63	3.42	6.88	2.62
C*M									
A1	38.31	30.14	8.36cd	4.72b	6.96	1.86b	0.94c	3.26b	1.22c
A2	37.69	30.36	4.42de	6.4b	7.25	1.98b	1.86c	3.53ab	1.64bc
A3	29.15	27.52	22.95a	4.38b	5.29	1.95b	1.25c	2.98b	1.3c
A4	27.28	29.69	18.13b	5.17b	4.92	3.57a	2.18abc	3.42ab	1.36c
B1	36.11	26.89	2.82e	11.08a	5.82	3.42a	2.96ab	3.67ab	2.44a
B2	41.00	28.70	4.49cde	5.78b	5.65	2.64ab	1.72bc	2.97b	1.76bc
B3	35.42	24.17	8.87c	9.53a	5.05	3.23a	3.19a	4.09a	2.13ab
B4	31.79	26.45	18.33b	4.76b	5.85	2.05b	1.24c	3.46ab	1.25c
ANOVA table (LSD protected, $P \leq 0.05$)									
Cultivar	0.047	0.090	<.0001	0.001	0.408	0.098	0.040	0.207	0.002
Amendment	0.001	0.417	<.0001	0.023	0.350	0.629	0.708	0.710	0.067
C*M	0.198	0.971	0.001	0.001	0.501	0.007	0.012	0.046	0.014

Table S3. The related abundance (percentage of reads) and interaction effect analysis of bacteria at the genus level. A and B were representing the Caoyou1 and Baiyan2 oat cultivars, respectively. CK (A1 and B1) was negative control; F (A2 and B2) was bio-fertilizer treatment; R (A3 and B3) was rotten straw treatment; RF (A4 and B4) was bio-fertilizer + rotten straw treatment. Values were represented as means ± SEs, and the different small letters within each column of cultivar (C), amendment (M) and C*M means significantly differences at 0.05 level based on ANOVA test.

	Bacillus	Pseudarthrobacter	Azotobacter	Sphingomonas	Massilia	Planomicrobium	Nocardioides	Pseudoxanthomonas	Pseudomonas	Microvirga
Cultivar (C)										
Caoyou1(A)	8.52a	10.23a	0.49b	3.39	1.64a	2.23a	2.12	0.89	0.66b	1.05
Baiyan2(B)	5.54b	7.35b	1.94a	3.35	0.82b	1.30b	2.03	0.88	0.91a	1.17
Amendment (M)										
CK	5.35b	16.11	2.28ab	7.45	3.05b	1.71c	4.72a	1.72	1.6ab	2.00
F	3.24b	14.70	3.95a	7.59	4.45a	2.05c	5.62a	1.64	2.13a	2.45
R	22.16a	18.04	2.38ab	6.32	1.20c	4.67b	3.29b	2.33	1.46bc	2.17
RF	25.51a	21.48	1.11b	5.60	1.14c	5.72a	2.97b	1.40	1.06c	2.28
C*M										
A1	4.08cd	11.05	0.83bc	3.67	1.96b	1.25c	2.08	0.90	0.87b	1.09
A2	1.49d	8.13	0.26c	4.21	3.46a	1.06cd	3.04	0.84	0.75bc	1.08
A3	16.21a	10.76	0.65bc	3.03	0.64c	3.48a	1.64	1.10	0.58bc	1.07
A4	12.31b	10.97	0.22c	2.64	0.52c	3.15ab	1.71	0.74	0.42c	0.99
B1	1.27d	5.06	1.45bc	3.70	1.09c	0.45d	2.64	0.82	0.73bc	0.91
B2	1.75d	6.57	3.69a	3.39	0.99c	1cd	2.59	0.80	1.39a	1.37
B3	5.95c	7.20	1.73b	3.30	0.56c	1.19c	1.65	1.23	0.87b	1.10
B4	13.2b	10.50	0.89bc	2.96	0.62c	2.58b	1.26	0.66	0.64bc	1.30
ANOVA table (LSD protected, $P \leq 0.05$)										
Cultivar	0.001	0.007	<.0001	0.951	<.0001	<.0001	0.725	0.911	0.013	0.142
Amendment	<.0001	0.090	0.038	0.059	<.0001	<.0001	<.0001	0.231	0.006	0.303
C*M	<.0001	0.203	0.017	0.429	<.0001	0.002	0.135	0.903	0.034	0.152

Table S4. Proportion of variance explained by environmental variables determined by RDA based on the soil 16S OTUs matrix. The parameters were: GY, grain yield; FGY, fresh grass yield; DGY, dry grass yield; SW, soil water content; Salt, soil salt content; AK, soil available potassium; AP, soil available phosphorus; AN, soil inorganic-nitrogen; Cat., soil catalase activity; ALP, soil alkaline phosphatase activity; Ure., soil urease activity and Suc., soil sucrase activity.

Variables	R^2	Pr(>r)
GY	4.5125	**0.002**
FGY	4.0917	**0.003**
DGY	1.8873	0.077
SW	1.6265	0.12
pH	2.052	0.065
Salt	2.5582	**0.026**
AK	3.2773	**0.007**
AP	2.2879	**0.043**
AN	1.4127	0.177
ALP	1.5597	0.115
Cat.	1.327	0.193
Ure.	0.9228	0.483
Suc.	0.913	0.454

Table S5. Pearson correlation analysis (* $P < 0.05$, ** $P < 0.01$, *** $P < 0.001$) between soil properties and yields in all treatments. The parameters were: GY, grain yield; FGY, fresh grass yield; DGY, dry grass yield; SW, soil water content; Salt, soil salt content; AK, soil available potassium; AP, soil available phosphorus; AN, soil inorganic-nitrogen; Cat., soil catalase activity; ALP, soil alkaline phosphatase activity; Ure., soil urease activity and Suc., soil sucrase activity.

Parameters	SW	pH	Salt	AK	AP	AN	ALP	Cat.	Ure.	Suc.
GY	0.58**	-0.65***	0.73***	0.48*	0.64***	0.68***	0.17ns	0.13ns	-0.46*	-0.11ns
FGY	0.19ns	-0.33ns	0.19ns	-0.11ns	0.1ns	0.14ns	0.1ns	-0.05ns	-0.03ns	-0.27ns
DGY	0.38ns	-0.34ns	0.39ns	0.29ns	0.58**	0.35ns	0.39ns	-0.03ns	-0.17ns	-0.33ns

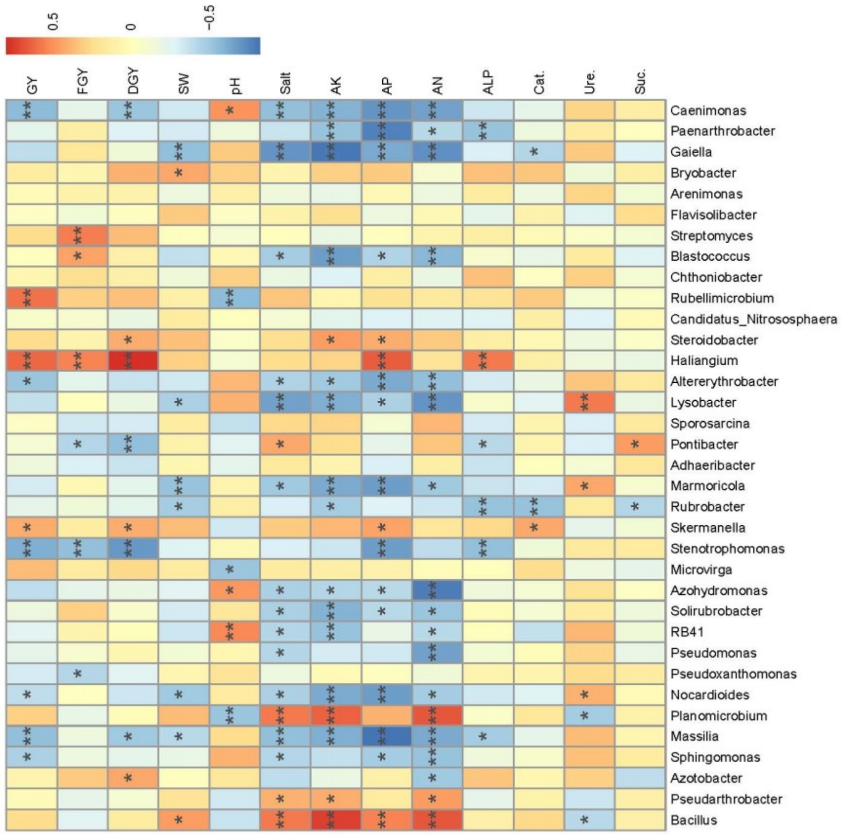

Figure S1. Spearman's rank-order correlation of bacterial genus, soil environments and yields where "r" value is between −1 and 1, r<0 is negative correlation, r>0 is positive correlation. Marked *, ** and *** indicated significance test p<0.05, p<0.01and p<0.001, respectively. the parameters were: GY, grain yield; FGY, fresh grass yield; DGY, dry grass yield; SW, soil water content; Salt, soil salt content; AK, soil available potassium; AP, soil available phosphorus; AN, soil inorganic-nitrogen; Cat., soil catalase activity; ALP, soil alkaline phosphatase activity; Ure., soil urease activity and Suc., soil sucrase activity.
